# Supplementary material for: Malaria Severity in the Elimination Continuum: A Retrospective Cohort Study between Beitbridge and Lupane Districts in Zimbabwe, 2021–2023
Source: Int J Environ Res Public Health. 2024 Jul 4;21(7):877. doi: 10.3390/ijerph21070877 (PMC11276829; doi:10.3390/ijerph21070877)
Supplement: Supplementary file 1 [file ijerph-21-00877-s001.zip › ijerph-3039892-supplementary.pdf]

Table S1: Operational Definition

(i) Dependent variable

No.	Variable	Definition	Register/Form	Scale	Category	Code
*DV	Malaria Severity	Malaria severity as defined according to the WHO guidelines as below Uncomplicated Malaria: This refers to cases of malaria where there are no clinical or laboratory signs of severe disease. Symptoms typically include fever, chills, headache, muscle aches, and fatigue. Severe Malaria: Severe malaria is characterized by the presence of one or more clinical or laboratory features indicating a risk of complications or organ dysfunction	Enrolment Notification Investigation	Ordinal	Uncomplicated malaria case<br>Severe malaria case	0<br>1

(ii) Independent variable

No.	Variable	Definition	Register/Form	Scale	Category	Code
*IV	Travel History Exposure	Individuals' history of travel to regions where malaria is prevalent as following: YES (If *Yes* to Within-country or If *Yes* to Foreign travel or If *Yes* wihin 6 weeks Pre-diagnosis) and NO (If *No* to Within-country or If *No* to Foreign travel or If *No* to within 6 weeks Pre-diagnosis)	Notification	Ordinal	No<br>Yes	0<br>1
1	Within-Country Travel	Malarious Area within-CountryTravel	Notification	Ordinal	No<br>Yes	0<br>1
2	Foreign Travel	Travel to places outside the country of residence in the within 6 weeks	Notification	Ordinal	No<br>Yes	0<br>1
3	Pre-diagnosis Travel	Travel within 6 weeks prior to diagnosis	Notification	Ordinal	No<br>Yes	0<br>1

***DV dependent variable** ***IV main independent variable**

(iii) Confounding variables

A. Socio-demographic variables

No.	Variable	Definition	Register/Form	Scale	Category	Code
1	District	District of residence during the study period	Notification<br>Investigation<br>Follow-up	Nominal	Lupane<br>Beitbridge	0<br>1
2	Year	Year of diagnosis of each case by the health facility	Notification<br>Investigation<br>Follow-up	Ordinal	2021<br>2022<br>2023	0<br>1<br>2
3	Age group	Broader age group (in years)	Notification<br>Investigation	Ordinal	< 5 years<br>5 years +	0<br>1
4	Age subgroup	Narrowed age group (in years)	Notification<br>Investigation<br>Follow-up	Ordinal *(intervals between thecategories are not equal)*	<5<br>5 - 14<br>15 - 19<br>20 - 64<br>65 +	0<br>1<br>2<br>3<br>4
5	Gender	Sex	Notification<br>Investigation<br>Follow-up	Nominal	female<br>male	0<br>1
6	Residence	Area of residence	Notification<br>Investigation<br>Follow-up	Nominal	Urban<br>Rural	0<br>1
7	Visitor	Individual received a visitor in the last six weeks	Notification<br>Investigation	Ordinal	No<br>Yes	0<br>1
8	Occupation	Individual's occupation status	Notification	Ordinal	Minor<br>Student<br>Unemployed<br>Employed	0<br>1<br>2<br>3

B. Malaria prevention Practices

No.	Variable	Definition	Register/Form	Scale	Category	Code
1	Prompt treatment	Treatment within 24 hours of fever onset	Notification<br>Investigation<br>Follow-up	Ordinal	Within 24 hours<br>After 24 hours	0<br>1
2	Malaria parasite	Type of plasmodium infections detected by laboratory tests	Enrolment<br>Notification<br>Investigation	Nominal	Other<br>Malariae<br>Falciparum	0<br>1<br>2
3	Contact	Malaria Contact Classification	Enrolment<br>Notification<br>Investigation	Ordinal	Asymptomatic<br>Symptomatic<br>Index	0<br>1<br>2
4	LLINs Ownership	LLINs in household	Investigation	Ordinal	Available<br>Unavailable	0<br>1
5			Enrolment	Ordinal	Used	0

	LLINs in previous night	LLINs use previous night	Investigation		Not used	1
6	LLINs use	LLINs ownership and usage	Investigation	Ordinal	Available and Used	0
					Available and Not used	1
					Unavailable and Not used	2
7	Slept Outdoors	Slept Outdoors during the night within 6 weeks	Notification	Ordinal	No	0
					Yes	1

Code 0 serves as the non-exposure or reference category for the binary dummy variables

Table S2: Overall Descriptive Results

Variable	Beitbridge		Lupane		Total	
	n	%	n	%	n	%
Age group						
<5years	77	6	122	10	199	8
5 years +	1130	94	1085	90	2215	92
Total	1207	100	1207	100	2414	100
Median ± SD					18 ± 16	
Sex						
Female	670	55	279	23	949	39
Male	537	45	928	77	1465	61
Total	1207	100	1207	100	2414	100
Sex ratio (F/M)					1:1.5	
Travel History						
No	1056	87	1056	87	2112	88
Yes	151	13	151	13	302	12
Total	1207	100	1207	100	2414	100
Occupation						
Minor	371	31	425	35	796	33
Student	132	11	161	13	293	12
Unemployed	286	24	161	13	447	19
Employed	418	34	460	39	878	36
Total	1207	100	1207	100	2414	100
Had visitor(s)						
No	232	19	580	48	812	34
Yes	975	81	627	52	1602	66
Total	1207	100	1207	100	2414	100
Residence						
Urban	491	41	609	51	1100	46
Rural	716	59	598	49	1314	54
Total	1207	100	1207	100	2414	100
Prompt treatment						
Within 24 hours	848	70	720	60	1568	65
After 24 hours	359	30	487	40	846	35
Total	1207	100	1207	100	2414	100
Malaria parasite						
Other	26	2	30	3	56	2
Malariae	103	9	159	13	262	11
Falciparum	1078	89	1018	84	2096	87
Total	1207	100	1207	100	2414	100
Malaria contact						
Asymptomatic	12	1	2	0.2	14	1
Symptomatic	228	19	185	15	413	17
Index	967	80	1020	85	1987	82
Total	1207	100	1207	100	2414	100
LLINs ownership						
No	491	41	540	45	1031	43
Yes	716	59	667	55	1383	57
Total	1207	100	1207	100	2414	100
LLINs use (previous night)						
No	838	69	778	64	1616	67
Yes	369	31	429	36	798	33

Total	1207	100	1207	100	2414	100
LLINs use						
Available and used	369	30	429	35	798	33
Available but no use	347	29	238	20	585	24
None	491	41	540	45	1031	43
Total	1207	100	1207	100	2414	100
Slept outdoors						
No	954	79	598	49	1552	64
Yes	253	21	609	51	862	36
Total	1207	100	1207	100	2414	100

Table S3: Bivariate Results for Beitbridge District

Variable	Malaria Severity						Chi-square	Bivariate Analysis	
	Uncomplicated		Severe		Total				
	n	%	n	%	n	%	P-value	RR; 95% CI	P-value
Travel History									
No	149	99	2	1	151	100		1	
Yes	937	89	119	11	1056	100	N/A	0.106(0.026;0.432)	<0.001*
Age group									
<5years	61	79	16	21	77	100			
5 years +	1025	91	105	9	1130	100	0.001*	0.39(0.22;0.70)	0.002*
Sex									
Female	688	99	2	1	670	100		1	
Male	418	78	119	22	537	100	N/A	95.1(23.4;386)	<0.001*
Occupation									
Minor	335	90	36	10	371	100		1	
Student	116	88	16	12	132	100		1.28(0.69;2.40)	0.434
Unemployed	266	93	20	7	286	100	0.177	0.70(0.30;1.24)	0.219*
Employed	369	88	49	12	418	100		1.24(0.78;1.95)	0.362
Had visitor(s)									
No	213	92	19	8	232	100		1	
Yes	873	90	102	10	975	100	0.300	1.31(0.79;2.19)	0.302
Residence									
Urban	444	90	47	10	491	100		1	
Rural	642	90	74	10	716	100	0.665	1.09(0.74;1.60)	0.665
Prompt treatment									
Within 24 hrs	813	96	35	4	848	100		1	
After 24 hours	273	76	86	24	359	100	<0.001*	7.32(4.83;11.1)	<0.001*
Malaria parasite									
Other	26	100	0	0	26	100	N/A	N/A	N/A
Malariae	100	97	3	3	103	100	N/A	N/A	N/A
Falciparum	960	89	118	11	1078	100	N/A	N/A	N/A
Malaria contact									
Asymptomatic	12	100	0	0	12	100	N/A	N/A	N/A
Symptomatic	228	100	0	0	228	100	N/A	N/A	N/A
Index	846	88	121	12	967	100	N/A	N/A	N/A
LLINs use									
Available-used	357	97	12	3	369	100		1	
Available-not use	318	92	29	8	347	100		2.71(1.36;5.41)	<0.001*
None	411	84	80	16	491	100	<0.001*	5.79(3.11;10.8)	<0.001*
Slept outdoors									
No	933	98	21	2	954	100		1	
Yes	153	61	100	39	253	100	<0.001*	29.0(17.6;48.0)	<0.001*

Table S4: Bivariate Results for Lupane District

Variable	Malaria Severity						Chi-square	Bivariate Analysis	
	Uncomplicated		Severe		Total				
	n	%	n	%	n	%	P-value	RR; 95% CI	P-value
**Travel History**									
Travelled	148	98	3	2	151	100		1	
Not Travelled	808	77	248	23	1056	100	N/A	0.066(0.021;0.209)	<0.001*
**Age group**									
<5years	58	48	64	52	122	100		1	
5 years +	898	83	187	17	1085	100	<0.001*	0.19(0.13;0.29)	<0.001*
**Sex**									
Female	196	70	83	30	279	100		1	
Male	760	82	168	18	928	100	<0.001*	0.52(0.38;0.71)	<0.001*
**Occupation**									
Minor	320	75	105	25	425	100		1	
Student	128	80	33	20	161	100		0.79(0.51;1.22)	0.28
Unemployed	111	69	50	31	161	100	<0.001*	1.37(0.92;2.05)	0.12*
Employed	397	86	63	14	460	100		0.48(0.34;0.68)	0.00*
**Had visitor(s)**									
No	534	92	46	8	580	100		1	
Yes	422	67	205	33	627	100	<0.001*	5.64(4.00;7.96)	<0.001*
**Residence**									
Urban	534	88	75	12	609	100		1	
Rural	422	71	176	29	598	100	<0.001*	2.97(2.20;4.00)	<0.001*
**Prompt treatment**									
Within 24 hrs	564	78	156	22	720	100		1	
After 24 hours	392	81	95	19	487	100	0.36	0.88(0.66;1.17)	0.37
**Malaria parasite**									
Other	29	97	1	3	30	100		1	
Malariae	141	89	18	11	159	100	N/A	3.70(0.48;28.8)	0.211*
Falciparum	786	77	232	23	1018	100		8.56(1.16;63.2)	0.035*
**Malaria contact**									
Asymptomatic	2	100	0	0	2	100	N/A	N/A	N/A
Symptomatic	170	92	15	8	185	100	N/A	N/A	N/A
Index	784	77	236	23	1020	100	N/A	N/A	N/A
**LLINs use**									
Available-used	412	96	17	4	429	100		1	
Available-not use	169	71	69	29	238	100	<0.001*	9.90(5.65;17.3)	<0.001*
None	375	69	165	31	540	100		10.7(6.35;17.9)	<0.001*
**Slept outdoors**									
No	488	82	110	18	598	100		1	
Yes	468	77	141	23	609	100	0.042*	1.34(1.01;1.77)	0.042*

Table S5: Bivariate Selection Results

Variables	Beitbridge District		Lupane District	
	**P-value**	**Decision**	**P-value**	**Decision**
Travel History	<0.001*	To multivariate	<0.001*	To multivariate
Age group	0.004*	To multivariate	<0.001*	To multivariate
Sex	<0.001*	To multivariate	<0.001*	To multivariate
Occupation	0.161*	To multivariate	<0.001*	To multivariate
Had visitor(s)	0.290	Important (proceed)	<0.001*	To multivariate
Residence	0.664	Important (proceed)	<0.001*	To multivariate
Prompt treatment	<0.001*	To multivariate	0.363	Important (proceed)
Malaria species	n/a	Removed	0.035*	To multivariate
Malaria contact	n/a	Removed	n/a	Removed
LLINs use	<0.001*	To multivariate	<0.001*	To multivariate
Slept outdoors	<0.001*	To multivariate	0.041*	To multivariate

Statistically significant Chi-square ($p \leq 0.25$*) Omnibus Tests

Table S6: Overall Multivariate Results

Variables	Category	N (%)	Malaria Severity		Multivariate Regression	
			Uncomplicated	Severe	RR; 95% CI	P-value
Travel History	No	2112(88)	1745(85)	367(98)	1	
	Yes	302(12)	297(15)	5(2)	0.18 (0.05;0.70)	0.013
District	Lupane	1207(50)	956(47)	251(67)		
	Beitbridge	1207(50)	1086(53)	121(33)	0.37(0.28;0.51)	<0.001
					80% (>10%)*	
Age group	<5years	166(8)	119(6)	80(22)	1	
	5 years +	2215(92)	1923(94)	292(78)	0.23(0.15;0.35)	<0.001
Mean ± SD	*23 ± 15.6*				84 % (>10%)*	
Median ± SD	*18 ± 15.6*					
Gender	Female	949 (39)	864 (42)	85(23)	1	
	Male	1465(61)	1178 (57)	287(77)	2.77(2.01;3.81)	<0.001
Sex ratio	*1:1.5(F/M)*				85 % (>10%)*	
Occupation	Minor	796(33)	655(32)	141(38)	1	
	Student	293(12)	244(12)	49(13)	1.40(0.89;2.19)	0.149
	Unemployed	447(19)	377(19)	70(19)	1.50(1.00;2.27)	0.052
	Working	878(36)	766(37)	112(30)	1.17(0.82;1.67)	0.397
					85 % (>10%)*	
Had visitor(s)	No	812(34)	747 (37)	65 (18)	1	
	Yes	1602(66)	1295(63)	307(82)	5.45(3.81;7.80)	<0.001
					82% (>10%)*	
Residence	Urban	812(34)	978(48)	122(33)	1	
	Rural	1602(66)	1064(52)	250 (67)	1.62(1.21;2.17)	0.001
					89% (>10%)*	
Prompt treatment	Within 24hrs	1568(65)	1377 (67)	191 (51)	1	
	After 24hrs	846 (35)	665 (33)	181 (49)	2.04(1.53;2.70)	<0.001
Mean ± SD	*2.4± 2.30*				81% (>10%)*	
Malaria species	Other	56 (2)	53 (3)	1 (0.3)	1	
	Malariae	262 (11)	241 (12)	21 (6)	0.53(0.05;5.64)	0.602
	Falciparum	2096 (87)	1746 (85)	350 (94)	1.02(0.10;10.02)	0.996
					86% (>10%)*	
Malaria contact	Asymptomatic	14 (1)	14 (0.7)	0 (0)	1	
	Symptomatic	413 (17)	398 (20)	15 (4)	--	--
	Index	1987(82)	1630 (80)	357 (96)	--	--
LLINs use	Available used	798(33)	769(37)	29(8)	1	
	Available unused	585(24)	487(24)	98(26)	6.34(3.95;10.18)	<0.001
	None	1031(43)	786(39)	245(66)	13.48(8.66;21.00)	<0.001
					70.0% (>10%)*	
Slept outdoors	No	1552(64%)	1421 (70)	131(35)	1	
	Yes	862 (36%)	621 (30)	241(65)	5.11(3.82;6.85)	<0.001
					76%> (10%)	
Travel*Sex	--	--	--	--	0.04(0.01;0.27)	0.001*
